# Improved Thermal and Electrical Properties of P-I-N-Structured Perovskite Solar Cells Using ZnO-Added PCBM as Electron Transport Layer

**DOI:** 10.3390/ma17061376

**Published:** 2024-03-17

**Authors:** Younghun Jeong, Dongwoon Han, Seongtak Kim, Chan Bin Mo

**Affiliations:** Functional Materials and Components Group, Gwanwon Technology Application Division (Functional Materials), Korea Institute of Industrial Technology, Wonju 26336, Republic of Korea; yh.jeong@fespv.com (Y.J.);

**Keywords:** perovskite solar cells, electron transport layer, thermal conductivity, electrical property

## Abstract

Not only can perovskite solar cells be exposed to high temperatures, up to 80 °C, depending on the operating environment, but absorbed energy is lost as heat, so it is important to have thermal stability for commercialization. However, in the case of the recently reported p-i-n structure solar cell, most of the electron and hole transport layers are composed of organic materials vulnerable to heat transfer, so the light absorption layer may be continuously exposed to high temperatures when the solar cell is operated. In this study, we attempted to improve the thermal conductivity of the electron transport layer using phenyl-C61-butyric acid methyl ester (PCBM) containing zinc oxide (ZnO). As a result, the thermal conductivity was improved by more than 7.4% and 23.5% by adding 6.57vol% and 22.38vol% of ZnO to PCBM, respectively. In addition, the insertion of ZnO resulted in changes in the electron transport behavior and energy level of the electron transport layer. As a result, it was confirmed that not only could the temperature stability of the perovskite thin film be improved, but the efficiency of the solar cell could also be improved from 14.12% to 17.97%.

## 1. Introduction

Perovskite (PVSK) is close to commercialization as it has been announced that its efficiency is comparable to crystal silicon solar cells, which account for 90% of the market share [1]. PVSK solar cells not only have the advantages of inexpensive, tunable band gap and long diffusion length but can also be used in a variety of applications, such as semitransparent solar cells [2,3,4]. In addition, due to its high efficiency in low light, it is receiving attention as an energy source using indoor light, and the launch of solar cell products for indoor light has recently been reported [5,6]. For perovskite solar cells to be commercialized as solar cells for power generation equivalent to silicon solar cells, the most important factors are efficiency, lifespan, and cost [7]. The efficiency is close to that of commercial solar cells, and the cost is competitive due to the advantages of thin film solar cells, but stability still remains an issue to be solved [8,9,10,11]. Another advantage of perovskite solar cells is that they can produce flexible solar cells, which cannot be produced with silicon solar cells [12,13]. In particular, p-i-n structure solar cells have the advantage of being manufactured through a low-temperature process using organic materials, contrary to n-i-p structures, which commonly use TiO_2_ and SnO_2_ as electron transport layers (ETLs) through high-temperature processes [14,15,16]. Therefore, studies have been reported on the development of various organic materials for the electron and hole transport layer (HTL) that can be applied to p-i-n structure solar cells and the production of flexible solar cells using roll-to-roll processes [17,18,19].

However, organic materials such as polytriarylamine(PTAA) and PCBM used as ETL in p-i-n structure solar cells are known to have very low thermal conductivity [20,21]. Since ETL and HTL have very low thermal conductivity, it is difficult to dissipate the heat generated from the solar cell. Although PVSK solar cells with improved stability using 2D PVSK and interfacial engineering have been reported [22,23,24,25], normally, perovskite materials have poor thermal stability, so it is necessary to improve heat dissipation performance by improving heat transfer in the ETL and HTL surrounding the light absorption layer. In addition, in order to apply solar cells to various applications, such as energy sources for human body-attachable medical devices and smart electronic devices, it is important to quickly remove internal heat through the heat dissipation design of the device. Recently, research results were published on improving the heat dissipation performance of solar cells by inserting thermally conductive fillers into the HTL [26,27]. However, there is little research on p-i-n structure perovskite solar cells using ETL, which has very low thermal conductivity.

In this study, in order to effectively dissipate heat that may be generated in p-i-n structure perovskite solar cells, we attempted to improve thermal conductivity by inserting heat transfer filler into the commonly used PCBM ETL. We used ZnO nanoparticles (NPs) in selecting the thermally conductive filler by considering the following two factors: (1) conducting heat without interfering with electrical conduction, (2) cost-effectiveness compared to other materials such as diamond, carbon nanotube aluminum nitrate, etc. As a result, a simple method was proposed to increase the power conversion efficiency (PCE) and thermal stability of PVSK by improving the heat dissipation characteristics of ETL.

## 2. Materials and Methods

**Figure 1:**
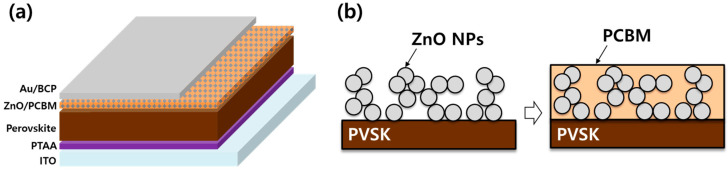
(**a**) Schematic configuration of p-i-n-structure PVSK solar cell, (**b**) sequential coating procedure for xwt%ZnO/PCBM film formation.

Device Fabrication The PVSK device is fabricated on indium tin oxide (ITO) glass. The ITO is cleaned with acetone, ethanol, and isopropyl alcohol (IPA) for 10 min, respectively. After that, the ITO is treated with UV ozone cleaner for 30 min. A PTAA solution diluted to 2 mg/mL in chlorobenzene (CB) is spin-coated on ITO at 6000 rpm for 30 s and annealed at 100 °C for 10 min. To prepare a perovskite precursor with a composition of 1.8 M (MAPbBr_3_)_0.08_(FAPbI_3_)_0.92_ (MA: methylammonium, FA: formamidinium, MAI, FAI from GreatCell Solar, Bomen, NSW, Australia), 763 mg of PbI_2_ and 53 mg of PbBr_2_ (TCI, Tokyo, Japan) were diluted in 1000 μL solution (dimethylformamide:dimethyl sulfoxide = 4:1) at 180 °C. The prepared solution was stirred at 60 °C by adding 16 mg, 285 mg, and 37 mg of MABr, FAI, and MACl, respectively. 120 μL of the precursor solution was spin-coated onto the ITO/PTAA substrate at 1000 rpm for 10 s and then at 5000 rpm for 30 s. During the second step, 500 μL of diethyl ether solution was quickly dripped. Then, the substrate was annealed at 120 °C for 20 min. ZnO with a size of 8–16 nm was prepared from Sigma Aldrich (zinc oxide nanoparticle ink, 2.5wt%). 0.01~1.0wt% ZnO NP dispersion diluted in isopropanol (IPA) is spin-coated on ITO/PTAA/PVSK at 5000 rpm for 20 s (Figure 1). The PCBM (Nano-C, Westwood, MA, USA) solution diluted to 20 mg/mL in CB was spin-coated at 2000 rpm for 60 s. Then, the bathocuproine solution diluted to 0.5 mg/mL in IPA is spin-coated at 4000 rpm for 30 s. Finally, a 100 nm Au layer was deposited by thermal evaporation. All materials not mentioned were purchased from Sigma-Aldrich Korea, Seoul, Republic of Korea).

Characterization A scanning electron microscope (SEM, SU5000, Hitachi, Tokyo, Japan) equipment was used to analyze the surface and cross-section of thin films. An ultraviolet photoelectron spectroscopy (UPS, K-alpha+, Therom Fisher Scientific, Waltham, MA, USA) was used to analyze the electron energy level of the xwt% ZnO/PCBM and ZnO films. Steady-state photoluminescence (PL) was performed using a single-mode diode laser (470 nm) and an inverted structure-type confocal microscope (MicroTime-200, Picoquant, Berlin, Germany) to investigate the charge transfer at PVSK/xwt% ZnO/PCBM interface. The amount of ZnO and PCBM were analyzed using inductively coupled plasma atomic emission spectroscopy (ICP-AES, Spectro Arcos, Spectro, Kleve, Germany). X-ray diffraction (XRD, Rigaku, SmartLab, Tokyo, Japan) observed the phases of the PVSK films using a Cu source. Analysis was performed in step mode at a scan rate of 3 degrees/min. An atomic force microscopy (AFM, Park SYSTEMs, Suwon, Republic of Korea) analysis was performed to measure the roughness of ZnO particles on the PVSK. An ultraviolet-visible-near infrared (UV-Vis-NIR, V-670 UV-VIS-NIR spectrophotometer, JASCO, Tokyo, Japan) was performed to investigate the optical bandgap of ZnO and xwt% ZnO/PCBM films. The I-V characteristics of the solar cells were measured using a Keithley 2400 source meter under an AM1.5G solar simulator (WXS-155S-10 class AAA, Wacom Denso, Fukaya, Japan) for light I-V. The light I-V curves were obtained using 200 ms delay times in the reverse scan direction (1.2 V to 0 V). Six solar cells manufactured for each condition were evaluated.

Thermal conductivity analysis A thermal conductivity of the xwt% ZnO/PCBM films was analyzed using a time-domain thermoreflectance (TDTR, Transometer^TM^, TMX Scientific, Richardson, TX, USA) method. The thermal conductivity (κ) was calculated using the equation:

(1) $$\kappa (W/mK)=\alpha \;({mm}^{2}/s)\times c\;\left(J/(g\cdot K)\right)\times \rho \;(g/{cm}^{3})$$

where, *α* is the thermal diffusivity, *c* is the heat capacity and ρ is the density of xwt% ZnO/PCBM films. Specific heat was measured using differential scanning calorimetry (DSC204, Netzsch, Burladingen, Germany). The density of the film was extracted proportionally according to the content ratio of PCBM and ZnO.

Heat Dissipation test The Heat dissipation behavior of xwt% ZnO/PCBM thin films was observed by forward-looking infrared (FLIR, E6xt, Teledyne FLIR, Wilsonville, OR, USA) images. The emissivity of the measurement parameter was fixed at a value of 0.98. First, the samples (ITO/PVSK/xwt%ZnO/PCBM) were placed on a hot plate set to 85 °C and heated until sufficient thermal equilibrium was reached. After the film was quickly moved into contact with the cu substrate, the temperature of the sample was observed using a thermal imaging camera. The thermal images of the samples were measured at 10 s intervals. The average temperature of the range of 7.5 × 7.5 mm^2^ in the center area of the thermal image was extracted as the representative temperature. For accurate relative comparison, the initial temperature was normalized, and the temperature drop due to heat dissipation was compared.

Thermal Stability Test The temperature was adjusted to 85 °C using a hot plate and checked using a thermocouple (k-type) and thermometer (Testo-922). The test was conducted in a homemade humidity control box (25% relative humidity, RH).

## 3. Results

**Figure 2:**
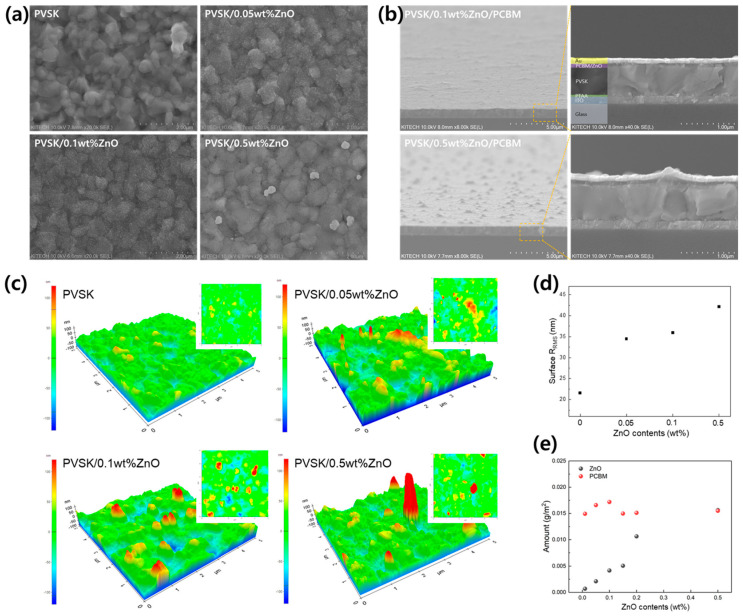
(**a**) Top view of xwt% ZnO on PVSK, (**b**) cross-sectional view of PVSK/xwt% ZnO/PCBM structures, (**c**) AFM, and (**d**) R_RMS_ with ZnO contents of xwt% ZnO on PVSK, (**e**) Amount of ZnO and PCBM coated on specific area with xwt% ZnO contents.

The top view of the xwt% ZnO on PVSK thin films is shown in Figure 2a. As the ZnO content coated on PVSK increases, it can be observed that ZnO in the form of white-colored dots is distributed on the PVSK surface. At a content of 0.05 and 0.1wt%, the ZnO-coated form appears to be very well dispersed. However, when the ZnO content increases to 0.5wt%, aggregates of approximately 200 nm are observed. It can also be revealed in the cross-sectional view in Figure 2b. In the case of the 0.1wt% ZnO structure, the surface is even, whereas in the case of 0.5wt% ZnO, it results in an uneven surface due to ZnO agglomerates on the surface. The root mean square surface roughness (R_RMS_) increased with increasing the ZnO content (Figure 2c,d). It is thought that as the concentration of ZnO increases, ZnO NPs agglomerate due to electrostatic attraction during coating. This is also clearly visible in the AFM results when 0.5wt% ZnO is coated. It is known that the ZnO NPs agglomerate depending on coating conditions [28]. In order to evenly apply ZnO to the PCBM using sequential coating, it seems important that the ZnO content does not exceed 0.5wt%. The actual coating amount of ZnO and PCBM in xwt% ZnO/PCBM films was analyzed according to the ZnO solution concentration. In Figure 2e and Table 1, it was confirmed that the amount of actually coated ZnO increased linearly as ZnO concentration increased. On the other hand, PCBM was coated with similar amounts regardless of the amount of ZnO coated. This means that even though the amount of coated ZnO increased, the PCBM did not completely cover the ZnO and did not become thick. This result corresponds to the cross-sectional observation result (Figure 2b). So, this sequential coating method can increase the ZnO content in PCBM without changing the coating thickness of the ZnO/PCBM film. When the ZnO content is increased to 0.5wt%, the ratio of ZnO and PCBM is about 1:1. On the other hand, the volume ratio is only 22.38%. In general, in order to effectively increase thermal conductivity through the connection of fillers in a heat-dissipation composite material with a filler and matrix structure, the filler content has to exceed a critical value. This is well known as the percolation effect [29]. According to reports, in the case of materials generally used as heat dissipation materials, 30vol% or more of filler is required for the percolation effect [30]. Although this sequential coating method for ZnO/PCBM film cannot insert fillers with a content of more than 30vol%, heat transfer can be expected through the already connected ZnO NPs while maintaining the PCBM thickness.

**Figure 3:**
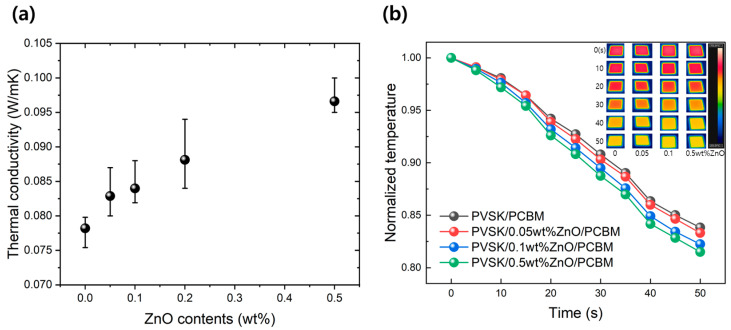
(**a**) Thermal conductivity of xwt% ZnO/PCBM films with ZnO contents, (**b**) Normalized temperature change with time and IR thermal images under heat dissipation test (insert).

Figure 3a shows the results of the thermal conductivity of the xwt% ZnO/PCBM according to the ZnO contents in the coating solution. The PCBM film with a thermal conductivity of 0.078 W/mK increased to 0.084 W/mK and 0.097 W/mK when the ZnO content was added at 0.1wt% and 0.5wt%, respectively. Based on the results of increases of 7.4% and 23.5% compared to PCBM, the coated ZnO NPs clearly improved the thermal conductivity of ZnO/PCBM film. The IR thermal images for evaluating the heat dissipation performance of the xwt% ZnO/PCBM thin film are shown in Figure 3b. The insert contains a thermal image of the thin film measured with a thermal imaging camera. As a result of tracking the temperature change of the specimen reduced by heat dissipation at 85 °C for 50 s, the ZnO-free PCBM showed a temperature decrease of 16.1% compared to the initial temperature, while the temperature of the 0.5wt% ZnO/PCBM decreased by 19.1%.

**Figure 4:**
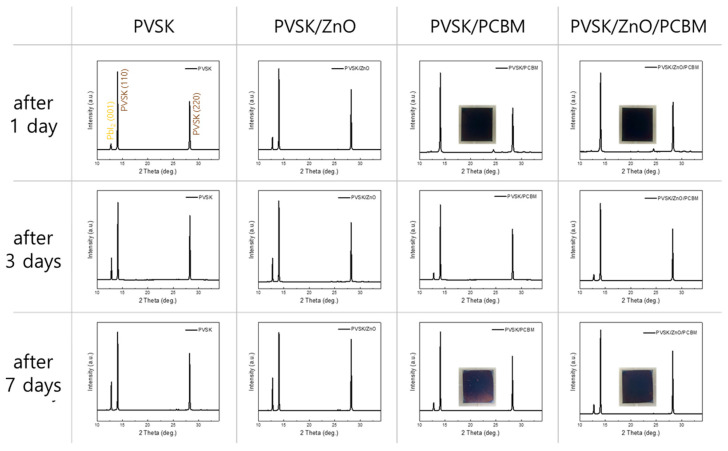
XRD results of PVSK, PVSK/ZnO, PVSK/PCBM and PVSK/ZnO/PCBM films after 1, 3 and 7 days of 85 °C/25%RH exposure. Insert images show the color of PVSK/PCBM and PVSK/ZnO/PCBM films during thermal stability test.

In order to observe changes in the thermal stability of PVSK film due to improved heat dissipation performance of ZnO/PCBM, XRD results were checked for 7 days at 85 °C/25%RH. The thermal decomposition was reduced in PVSK coated with ZnO/PCBM film (Figure 4). As a result of comparing PVSK and PVSK/ZnO, the thermal decomposition of PVSK was accelerated when ZnO was inserted compared to PVSK. According to previous literature, the thermal stability of PVSK deteriorates when ZnO and PVSK contact [31]. It is known that the oxygen of ZnO attracts the hydrogen of MA or FA in PVSK, promoting the decomposition of PVSK. On the other hand, when PCBM was coated on ZnO/PVSK, the thermal stability was significantly improved. Since the PCBM passivates the ZnO, it is thought to have no degradation effect on PVSK film, as reported in the literature [32]. Comparing PVSK/PCBM and PVSK/ZnO/PCBM, it can be seen that the thermal stability of PVSK in PVSK/ZnO/PCBM is slightly improved despite the presence of ZnO. As seen in the insert PVSK film image, no significant decomposition of PVSK was observed in the PVSK/ZnO/PCBM compared to PVSK/PCBM.

**Figure 5:**
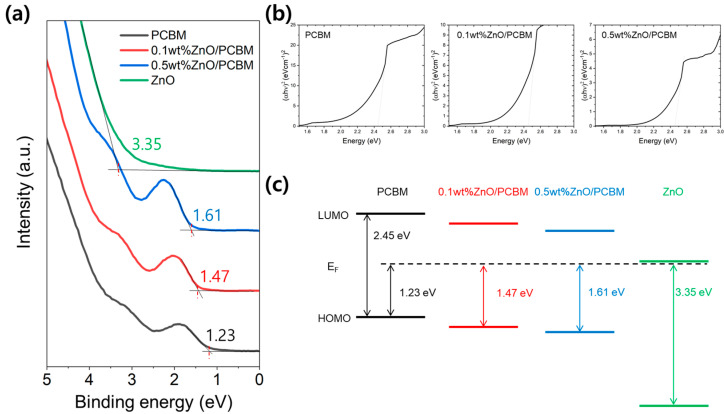
(**a**) UPS spectra, (**b**) optical band gap, and (**c**) energy-level changes of PCBM, Xwt% ZnO/PCBM, and ZnO.

The energy level of a thin film can be changed by even a very small amount of dopant or impurities [33,34]. It is necessary to confirm changes in the properties of the thin film due to the insertion of ZnO into the PCBM film. As a result of the energy level analysis in Figure 5, the LUMO of the PCBM film is located at 1.22 eV above the Fermi energy level (E_f_), and the HOMO is located at 1.23 eV below E_f_. While the optical bandgap did not change with the addition of a very small amount of ZnO content, LUMO and HOMO decreased as ZnO was mixed into the PCBM. This means that the energy level has changed due to the addition of ZnO, based on the fact that the HOMO and LUMO of ZnO have lower values than those of PCBM. Not only can electron transfer be facilitated by lowered LUMO by adding a small amount of ZnO in PCBM, but solar cells’ characteristics can be improved by delaying the recombination between electrons and holes through a hole-blocking effect due to lowered HOMO.

**Figure 6:**
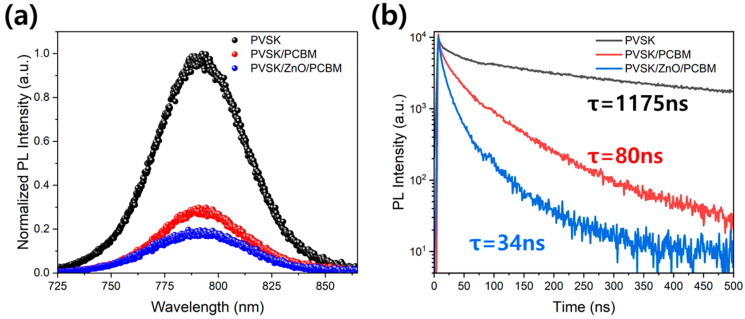
(**a**) PL spectra, and (**b**) Time-resolved photoluminescence decay spectra of PCBM, xwt% ZnO/PCBM and ZnO film.

The results of steady-state PL and TRPL analysis are shown in Figure 6 to confirm the improvement of the electron transfer effect with the addition of ZnO. As ZnO is added to PCBM, the PL intensity is lowered, and the lifetime is reduced from 80 ns to 34 ns. A decrease in PL intensity and lifetime indicates an increase in electron extraction and charge dissociation. Similar behaviors are also shown in previous literature [35,36]. Therefore, the energy level and electron extraction ability can be expected to improve just by adding a small amount of ZnO in PCBM.

**Figure 7:**
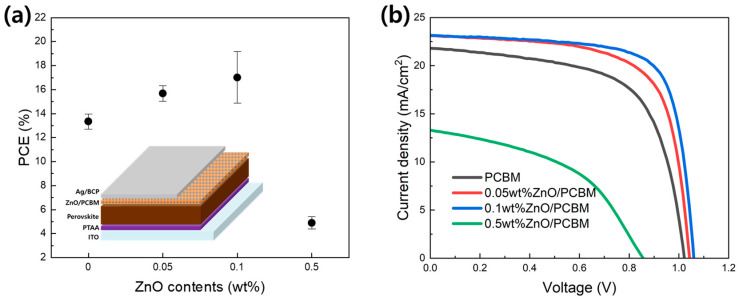
(**a**) Average PCE with ZnO contents in PCBM. The insert image is a schematic configuration of a p-i-n-structucture PVSK solar cell, (**b**) current density-voltage curves of representative conditions.

Figure 7 and Table 2 show the performance of the p-i-n structure PVSK solar cell using xwt% ZnO/PCBM as an electron transport layer. The PCE increased significantly from 14.12% to 17.97% as the ZnO content increased to 0.1wt%. When 0.5wt% was added, however, PCE rapidly decreased to 5.26%. Improving PCE was mainly due to increases in current density and fill factor. The increase in current density and fill factor is closely related to the electron transfer between PVSK and PCBM. The results of solar cell performance correspond to the improvement in electrical properties observed previously by adding ZnO to the PCBM. However, in the case of adding 0.5wt% ZnO, the solar cell characteristics are thought to have deteriorated due to the agglomeration of ZnO NPs. As shown in Figure 7b, not only the fill factor but also overall performance decreased. Therefore, manufacturing a ZnO/PCBM composite ETL using the sequential coating method can improve the thermal conductivity and electrical properties of the PVSK solar cells, but only an appropriate amount of control (0.1wt%) is required due to the agglomeration of ZnO NPs during the coating process. As a result, this method shows that PCE and thermal stability can be improved by inserting heat and electron transfer fillers into the ETL.

## 4. Conclusions

We incorporated ZnO into PCBM to improve not only heat transfer but also the electrical transfer of PVSK solar cells. The cooling test results of xwt% ZnO/PCBM showed that 0.1wt% ZnO insertion into the PCBM clearly affected the heat transfer of ETL. The addition of 6.57vol% and 22.38vol% ZnO to PCBM improved thermal conductivity by about 7.4% and 23.5%, respectively, compared to PCBM. As a result, the thermal stability of the PVSK has been improved because the heat of PVSK can be dissipated efficiently. In addition, the PCE improved to 17.97% (with 0.1wt% ZnO) compared to the control device (14.12%) due to the changes in energy level and enhanced electron transfer. This study suggests that considering the thermal conductivity of the electron transport layer when optimizing the device structure is important in terms of solar cell efficiency and long-term stability. In addition, a direction to overcome the low heat dissipation ability of p-i-n-structured PVSK solar cells using an organic ETL and HTL is presented.

## Figures and Tables

**Table 1:** Weight and volume amount of ZnO and PCBM in xwt% ZnO/PCBM films on PVSK.

ZnO Concentration (wt%)	Amount of ZnO (g/m^2^)	Amount of PCBM (g/m^2^)	Amount of ZnO in PCBM	
			wt%	vol%
0.01	0.00069	0.01493	4.44	1.33
0.05	0.00211	0.01658	11.28	3.56
0.10	0.00416	0.01718	19.48	6.57
0.15	0.00507	0.01497	25.31	8.97
0.20	0.01063	0.01511	41.30	16.98
0.50	0.01562	0.01549	49.79	22.38

**Table 2:** PVSK solar cells performance of p-i-n structure for various ETL structures.

ETL Structures	Voltage (V)	Current Density (mA/cm^2^)	Fill Factor (%)	PCE (%)
PCBM	1.02	21.73	63.60	14.12
0.05wt%ZnO/PCBM	1.04	23.00	68.81	16.51
0.1wt%ZnO/PCBM	1.06	23.03	73.58	17.97
0.5wt%ZnO/PCBM	0.86	13.25	46.34	5.26

## Data Availability

Data are contained within the article.
